# Mohs versus wide local excision: Highlighting the need to define optimal surgical strategies for hidradenocarcinoma

**DOI:** 10.1016/j.jdcr.2025.04.046

**Published:** 2025-06-16

**Authors:** Madalyn Bland South, Alfredo Siller, Matthew DaCunha, Stephen Tyring, Brett M. Coldiron

**Affiliations:** a Skin Cancer Center, Cincinnati, Ohio; b The University of Kentucky College of Medicine, Lexington, Kentucky; c Epiphany Dermatology, Overland Park, Kansas; d The University of Texas Health Science Center, Houston, Texas

**Keywords:** clear cell adenoma, cutaneous adenoma, dermatologic surgery, dermatopathology, hidradenocarcinoma, management of hidradenocarcinoma, micrographic surgery, Mohs, wide local excision

## Introduction

Hidradenocarcinoma, a rare malignancy of the sweat glands, comprises <1% of all cutaneous cancers and lacks standardized treatment guidelines.^1^^,^^2^ It is known for aggressive behavior, often metastasizing to regional lymph nodes, lungs, liver, bones, and brain, and has a 50% local recurrence rate and a 30% 5-year survival rate.^3^ Treatment typically involves wide excision, but chemotherapy, radiation, and Mohs micrographic surgery (MMS) are also utilized, depending on node involvement and other factors. We present 2 cases, highlighting the challenges in diagnosis and management and emphasizing the need for further research to establish optimal management strategies.

## Case report

### Case 1

**Fig 1:**
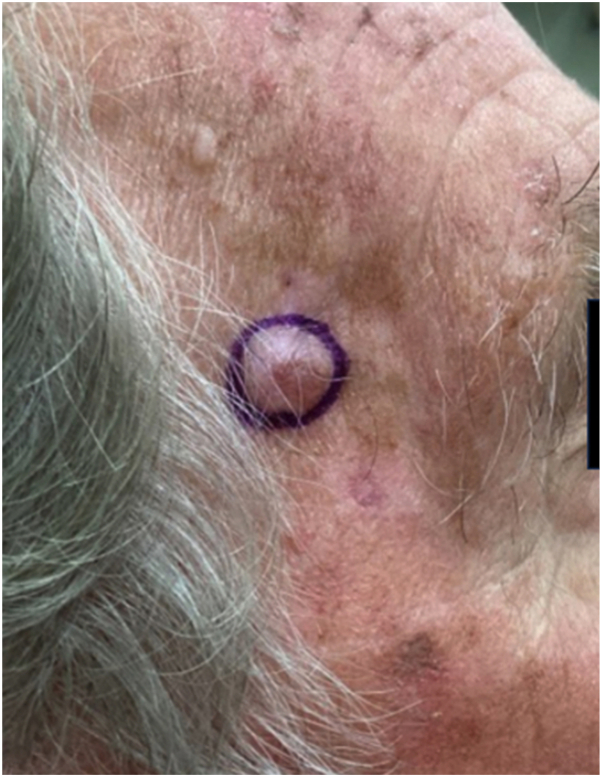
Demarcation of the lesion to be removed by Mohs micrographic surgery from the right temple of a 71-year-old man.

**Fig 2:**
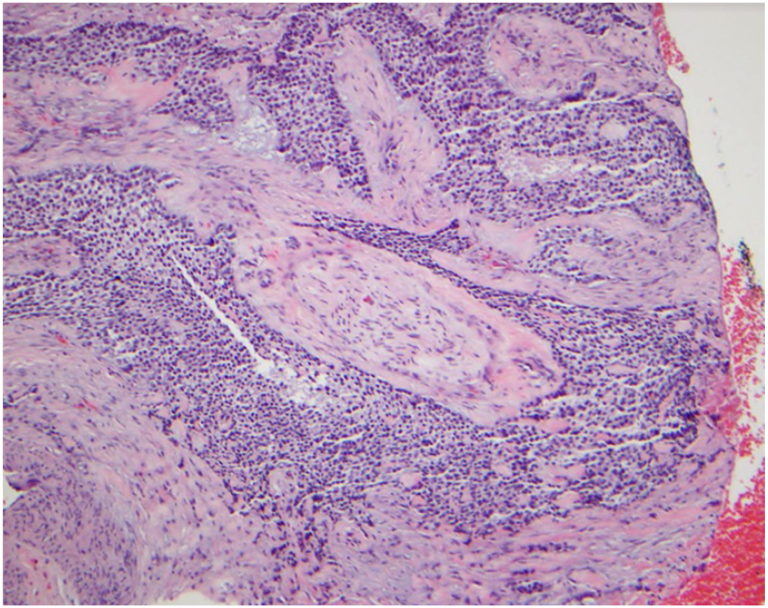
Hematoxylin stain of Mohs micrographic surgery-removed hidradenocarcinoma lesion. (Hematoxylin stain; original magnification: ×20).

A 71-year-old Caucasian male presented with a 1.1 × 1.1 cm red nodule on the right temple, growing over 2 months (Fig 1). The nodule had been steadily growing without symptoms such as bleeding, tenderness, scaling, drainage, or pruritus. Biopsy confirmed T1N0M0 hidradenocarcinoma (Fig 2). His physical exam was negative for regional lymphadenopathy, and medical and radiation oncology were consulted. Given the lack of clinical lymphadenopathy, size, and location being in a nonhair-bearing area, it was decided treatment would include MMS alone. The patient returned within 1 week of the biopsy sample for MMS, and it was cleared in 2 stages. He returned for his routine follow-ups at 1 month, 3 months, and 6 months, without any evidence of recurrence.

### Case 2

**Fig 3:**
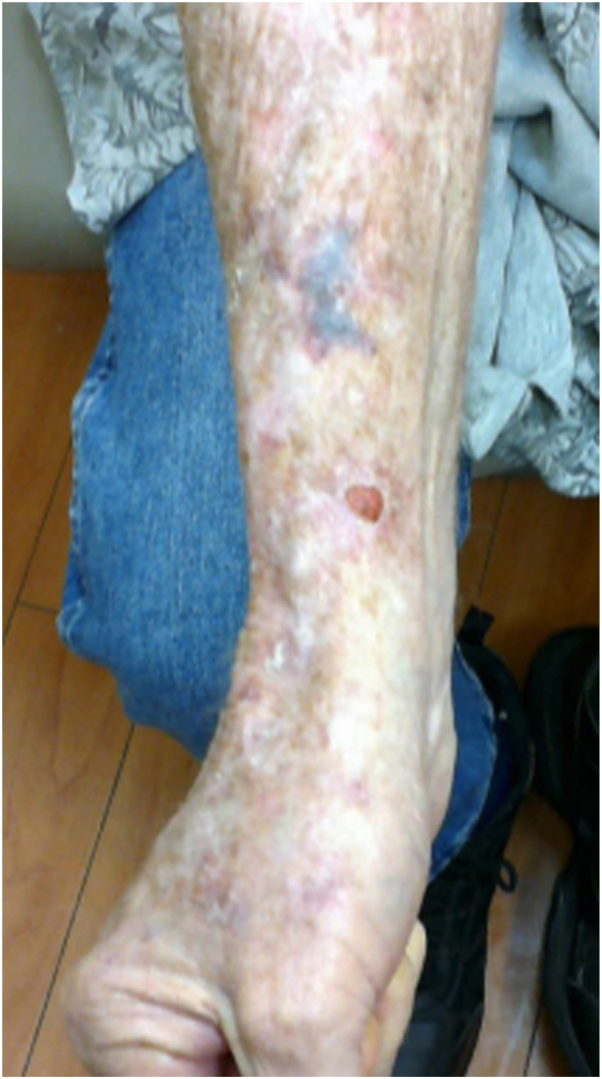
Initial biopsy site measuring 2.2 × 2.1 cm on the right wrist of an 83-year-old man.

**Fig 4:**
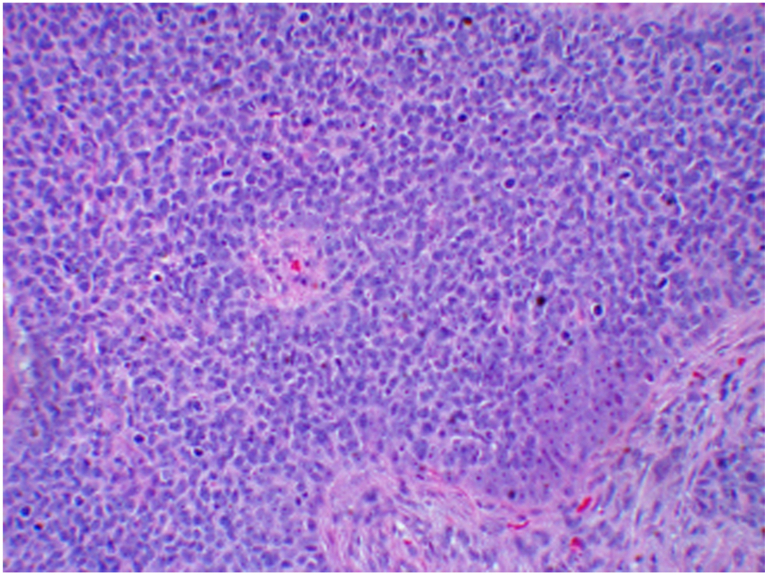
Hematoxylin stain of excised hidradenocarcinoma lesion. (Hematoxylin stain; original magnification: ×40).

An 83-year-old Caucasian male with a past medical history of diabetes mellitus, as well as melanoma and nonmelanoma skin cancer, presented with a 2.2 × 2.1 cm pearly telangiectatic nodule on the right wrist (Fig 3). The histological analysis of the nodule demonstrated initially ambiguous findings. The primary biopsy suggested basal cell carcinoma; however, the surface of another carcinoma such as a porocarcinoma or squamous cell carcinoma with basaloid differentiation could not be excluded. Excision was warranted both to ensure its complete removal and to provide any further residuum for histologic examination to confirm the diagnosis. Upon excision, further histology revealed hidradenocarcinoma (Fig 4). More specifically, the dermis presented a proliferation of fibroblasts aligned parallel to the skin surface interposed among linearly arranged, thickened collagen bundles and small blood vessels. Immunostain for multicytokeratin was positive, whereas chromogranin, neuron-specific enolase, and CD56 showed patchy nonspecific positivity. Cytokeratin 20 and synaptophysin were negative for neuroendocrine differentiation. Carcinoembryonic antigen and cytokeratin 7 failed to highlight definitive ducts. S100 was negative. The patient returned for routine follow-ups at 1, 3, and 6 months and showed no recurrence at the excision site.

## Conclusion

Diagnosing and treating hidradenocarcinoma remains challenging due to its rarity and resemblance to other cutaneous carcinomas, such as basal cell carcinoma.^1^, ^2^, ^3^, ^4^, ^5^, ^6^ Misdiagnosis can delay appropriate treatment, increasing the risk of local recurrence and metastasis.^5^ Our cases illustrate the importance of considering hidradenocarcinoma in the differential diagnosis of atypical skin lesions to prevent errors in the initial diagnosis.

Two different surgical techniques—MMS and wide local excision—are illustrated in our cases, highlighting the ongoing debate over optimal management. MMS has been associated with precise margin control and reduced recurrence rates, whereas wide local excision remains the standard approach for achieving negative margins.^4^^,^^5^^,^^7^^,^^8^ Despite surgical intervention, adjuvant therapies such as radiation or chemotherapy may be warranted in cases of nodal involvement or distant metastases.^4^^,^^9^

Further research in diagnostics, particularly immunohistochemistry, is crucial for distinguishing hidradenocarcinoma from more common malignancies.^6^^,^^10^ Given its high local recurrence rate of 50% and poor 5-year survival rate of 30%, rigorous surveillance with regular follow-ups is imperative to detect and manage recurrences early.^4^^,^^5^^,^^8^^,^^9^ Continued case reporting and collaborative studies with multidisciplinary approaches are essential to establish standardized treatment guidelines and improve long-term patient outcomes.^5^^,^^10^

## Conflicts of interest

None disclosed.
